# Schizophrenia and quality of life: how important are symptoms and functioning?

**DOI:** 10.1186/1752-4458-4-31

**Published:** 2010-12-08

**Authors:** Anna Galuppi, Maria Cristina Turola, Maria Giulia Nanni, Paola Mazzoni, Luigi Grassi

**Affiliations:** 1 Section of Child Neurology and Psychiatry, Children's Hospital A.Meyer - University of Florence, Italy; 2 Section of Psychiatry, University of Ferrara, Italy; 3 Unit of Clinical Psychiatry, Integrated Department of Mental Health and Drug Abuse, Local Health Agency, Ferrara, Italy; 4 Community Mental Health Centre, Integrated Department of Mental Health and Drug Abuse, Local Health Agency, Ferrara, Italy

## Abstract

**Objective:**

the relationship between Quality of life (QoL) and global functioning and symptoms in outpatients with Schizophrenia

**Method:**

The study was carried out on the outpatients with schizophrenia attending a Community Mental Health Centre in 2008. Each patient completed the WHO QoL Instrument - Brief and was administered the Brief Psychiatric Rating Scale-24 to assess psychiatric symptoms and the VADO Personal and social Functioning Scale to assess the level of functioning.

**Results:**

subjects showed an intermediate satisfaction on the overall QoL and health; these data can be juxtaposed to the national standard sample rates. QoL resulted positively associated to personal and social functioning, while it was negatively related to psychiatric symptoms.

**Conclusion:**

patients showed a fairly good satisfaction in regard to their QoL. The severity of psychiatric symptoms is one of the elements influencing QoL, together with personal and social functioning that plays a relevant role.

## Introduction

A specific interest regarding quality of life of patients with schizophrenia dates back to the de-institutionalisation process which took place in the 1960 s and 1970 s in several western countries [1,2]. In fact, as a result of mental health reforms, the effects of the shift of care from asylum to community health centres became a necessity for clinicians, researchers and health policy makers.

It was apparent that capturing psychopathological symptoms alone was not sufficient to reflect relevant outcomes. In particular, information on the social functioning and quality of life are regarded as essential for evaluating long-term outcomes.

Quality of life may be defined as a person's sense of wellbeing and satisfaction with his/her life circumstances, as well as a person's health status and access to resources and opportunities [3]. Clearly, such an outcome is of particular importance in order to develop treatments that can help individuals with schizophrenia to lead more fulfilling and satisfying lives [4].

Unfortunately, factors influencing QoL in schizophrenia are not well known. Studies addressing QoL for patients with schizophrenia and other severe mental illnesses have identified a number of important influential factors, such as social support [5], unmet needs, [6] and medication side effects [7].

However, most of the research examining factors affecting QoL has primarily focused on the impact of psychiatric symptoms. Some studies found from small to moderate relationships between psychiatric symptoms and QoL [8,9] while others presented findings suggesting that certain aspects of these concepts may be indistinguishable [10,11], particularly as far as negative symptoms and general psychopathology (eg, anxiety, depression) are concerned [4]. In recent years, several research groups have concluded that the so-called negative symptoms of schizophrenia are much more closely related to quality of life than positive symptoms [12,13].

In a study of 128 patients, Norman et al. [11] have examined the relationship of symptoms and level of functioning in schizophrenia to the quality of life: their results show that negative symptoms, level of functioning and positive symptoms all were related to the Quality of Life Scale.

On the contrary, in a study of 193 patients, Fitzgerald et al. [10] showed that subjectively reported life satisfaction was not related to positive or negative symptoms of schizophrenia but did correlate with depressive symptoms. A study in five European centres [6] measuring QoL and other patient and illness characteristics in a group of 143 outpatients with schizophrenia, found that patient's QoL is predicted mainly by anxiety and depression and by global functioning.

These variations among studies appear to be at least partially due to differences in the definition and measurement of QoL [14], given the complexity and heterogeneity of the concept of quality of life [15]. Some definitions of QoL refer to it as a multidimensional set of components consisting of a person's [1] satisfaction with his/her life as a whole, or general wellbeing; [2] observable social and material wellbeing, or objective QoL; [16] satisfaction with his/her social and material wellbeing, or subjective QoL; and [17] health and functional status, or health-related QoL [3]. Clarifying the relationship between psychiatric symptoms, global functioning and QoL represents an important step both in elucidating factors affecting QoL for individuals with schizophrenia and in understanding the utility of the concept of QoL for guiding future treatment development efforts [4].

Italy has implemented a decentralisation of its mental health services since 1978 with a major function in psychiatric care being placed in the Community Mental Health Centres (CMHC) that provide psychiatric integrated interventions in different settings, including outpatients clinics, and patients' own homes [18].

The aim of the present study was to assess the outcomes in all subjects with schizophrenic diagnosis attending a Community Mental Health Centre in Copparo (Ferrara-Italy) and to examine the relationships between quality of life, psychiatric symptoms and level of functioning.

## Materials and methods

### Study procedure

The present study was carried out from August to September 2008 in a Community Mental Health Centre, Copparo, of the Integrated Department of Mental Health in Ferrara, Emilia-Romagna Region, Northern Italy. Since the creation of the Department of Mental Health and the implementation of the psychiatric services after the 1978 reform in the Emilia-Romagna Region [18], community mental health centers have played a central role in delivering integrated care for patients with schizophrenia and their families.

Copparo Center is the health facility to which 37.803 inhabitants refer to, 19678 females and 18125 males; 4051 are under 18 and 10526 are over 65. Population is spread over 6 municipalities and mainly works in agricultural, commercial and industrial activities, in a flat countryside, well-supplied with facilities.

The Center is made up by an outpatient department and a day-hospital, both open eight hours a day on working days; it provides medical examinations and home consulting, drug therapies whenever needed, group therapies, individual and group rehabilitations activities, meetings with family members. Compulsory psychiatric hospitalizations for acute illnesses are made by the Psychiatric Unit of Ferrara Hospital, which is 18 km far, while voluntary hospitalizations for severe illnesses use both Ferrara and Lagosanto Hospital Units, 30 km far. Both units provide 15 beds, for the whole district, with 351463 caseload.

Rehabilitating hospitalizations, with projects from 1 to 3 months, are made in specific district facilities. There are three residences, providing 50 beds.

The Centre staff is composed by 6 psychiatric nurses, 2 psychiatrists, 1 psychologist and 1 social worker. The Centre deals with any sort of psychiatric illness, from reactive forms to psychoses, and supplies consulting at the local hospital and cooperates with family doctors and with the Department for Drug Abuse, which is in a different building. All subjects, even non-residents, have the right to receive free assistance, paying simply a money contribution. Subjects with a low income or with severe illnesses don't pay the money contribution. Schizophrenic subjects have totally free assistance.

Criteria for the inclusion in the study were: a) being outpatients at the CMHC; b) a diagnosis of schizophrenia according to the WHO-ICD-10 classification (F20.0 - F20:9) [19] c) age above 18 years.

Each patient was contacted by a research psychiatrist and a visit scheduled into the outpatient clinic of the CMH service, informed consent according to the Local Ethical Committee was gathered during the meeting.

A series of instruments were used to assess psychopathology, level of functioning and quality of life.

The World Health Organization Quality of Life - Brief (WHOQoL-Bref) was used to assess the patients' quality of life. The WHOQoL-BREF includes 26 items measuring the following domains: physical health, psychological health, social relationships, and environment. Two further items evaluate the individual's overall perception of quality of life and the individual's overall perception of their health. Domain scores are scaled in a positive direction (i.e. higher scores correspond to better quality of life). The average score of items within each domain is used to calculate the domain score. Mean scores are then multiplied by 4 in order to make domain scores comparable with the scores used in the WHOQOL-100. Where more than 20% of data is missing from an assessment, the assessment should be discarded. Where an item is missing, the average of other items in the domain is substituted. Where more than two items are missing from the domain, the domain score should not be calculated (with the exception of domain 3, where the domain should only be calculated if < 1 item is missing) [16].

The VADO Personal and Social Functioning Scale (FPS), which is a modified version of the Social and Occupational Functioning Assessment Scale (SOFAS) [17], was used to assess the patients' level of functioning in four main areas: work and/or socially useful activities; family, personal and social relationships; self-care; aggressive and destructive behaviours. Suicide risk is considered in the score only as much as suicidal ruminations may interfere with social-functioning. The FPS requires a brief and simple training, that is described in the VADO manual [20]. An evaluation is assessed according to the following levels: absent, slight, evident, marked, severe. The evaluation is then turned into a score from 0 to 100 (higher scores correspond to better functioning) according to VADO guide instructions.

In the present study, separate evaluations were carried out by two psychiatrists with training in the administration of the scale.

The Italian version of Brief Psychiatric Rating Scale (BPRS), in its 24-item 4.0 version [21,22] was used to assess psychiatric symptoms.

Each item is rated on a seven-point Likert scale (from 1 = no symptom to 7 = extremely severe symptom - range score = 24-168), yielding four factors: positive symptoms (items 9-12, 14-15, and 24), negative symptoms (items 13, 16-18, and 20), anxiety and depression (item 1-5, and 19), mania and hostility (items 6-8. and 21-23).

### Statistical Analysis

Statistic analysis was carried out using the SPSS 12.0 and Winstat for Excel statistic systems. Statistic procedure included the following survey: descriptive statistics, variation in answer distributions (frequencies), Pearson r Test, significance (p) test with a level of significance set at 0.05.

## Results

### Patients' characteristics

The study was proposed to all the 107 subjects of the Centre: 3 subjects, 2 women over 70, and a man who was 36 years'old refused to participate.

**Figure 1:**
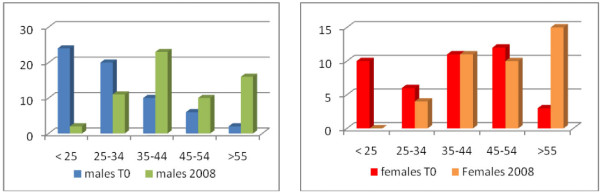
**Distribution by age groups**.

The characteristics of the subjects participating in the study are reported in Table 1. The sample consisted of 104 patients, of whom 62 males (59,6%) and 42 females (40,4%) with a mean age of 47 years (SD 13.5) The mean age of first contact with the CMH was 33.3 years (SD 13.6), and the duration of illness 13.4 years (SD 7.4). Distribution by age groups is shown in figure 1. 32 of the subjects (30.7%) were employed, 4 (3.8%) unemployed, 6 (5.7%) retired and 62 (59.6%) had a disability pension. 89 (85.6%) lived in their own house, while the rest lived in a group home, boarding home, or halfway house. 40 (38.5%) had a partner and 64 (61.5%) were single.

**Table 1:** Socio-demographic characteristics of the sample (n =104)

	Male	Female	Total
Number (%)	62 (59.6%)	42 (40.4%)	104

Age at 2008 (years)	44.9 ± 12.2	51.9 ± 12.5	47.0 ± 13.5

Age at T0* (years)	31.0 ± 11.4	37.9 ± 12.8	33,3 ± 13.6

Length of illness (years)	13.2 ± 7.1	13.5 ± 7.1	13.4 ± 7.37

Hospitalization rate Dd/year	18.0 ± 43.7	7.3 ± 62.8	

Outpatient intervention n/year	54.15 ± 52.8	39.1 ± 46.6	

Marital status	Single	64	61.5%
	Partner	40	38.5%

Housing	Private house	89	85,6%
	Group home/Boarding home/halfway house	15	14,4%

Occupation	Employed	32	30.7%
	unemployed	4	3.8%
	Disabled	62	59.6%
	Retired	6	5.7%

*T0 age = age when first arrived at Mental Health Department

2 subjects had no therapy, 20 subjects were under typic antipsychotics therapy, 54 atypics antipsychotics and 28 depot antipsychotics (26 of them were atypics).

The average hospitalization rate is 18.0 days per year of disease in males, 7.3 days per year in females.

On average, males had 54.15 ambulatory interventions, females 39.1; ambulatory interventions include examinations, conversations, group or family therapy meetings, with both nurse and doctor staff members.

32 males (51.6%) have attended a Day Center for at least a month, whereas only 12 females have (28.5%).

Outpatients in the past had 50 compulsory admissions in males, out of 358 admissions (13.97%), and 15 for females, out of 254 admissions (5.9%). Hospital admissions in the latest year were 8, divided among 6 persons, 5 males e 1 female: a man had three hospitalizations in a year, 5 subjects were undergoing a 6-months residential rehabilitation program.

### Quality of Life

Table 2 shows the mean scores related to every item of the four domains of the WHOQoL-BREF. In the first two items of the test, quality of life perception (Q1) recorded a 3.21 (SD 0.94) average score and 3.14 mean score on the perception of one's own health (Q2), corresponding to "neither satisfied nor dissatisfied". Total mean scores of the four domains show a higher level of satisfaction on physical and environmental health domain, while a lower level of satisfaction emerged in social relations domain.

**Table 2:** Perceived quality of life survey: mean scores on WHOQoL-BREF single items and mean scores on WHOQoL-BREF four domains.

Domain	Mean single item (SD)	Mean domain (SD)
Q1	3.21 (0.94)	

Q2	3.14 (0.98)	

Domain 1 (Physical)	3.26 (0.74)	22.8 (5.2)
Domain 2 (Psychological)	2.98 (0.52)	17.8 (3.2)
Domain 3 (Social)	2.43 (0.98)	7.2 (2.9)
Domain 4 (Environment)	3.06 (0.70)	24.4 (5.6)

Q1 = Overall perception of QoL; Q2 = Overall perception of Health

In table 3 the scores of the first 2 items and of the four domains are presented separately for men and women and by age groups (< 45 years and ≥ 45 years). Males' scores reveal a higher level of satisfaction than females' scores in all domains except domain 3 (social); males who were over 45 showed a wider quality of life perception (Q1) and perception of one's own health (Q2) than younger males, and show a better QoL in social and environmental relations; they show, however, a lower satisfaction in physical and psychological domain. Among the females, the group aging over 45 declared a lower satisfaction than the younger ones in all WHOQoL domains. Regarding physical health, differences between sexes and ages are statistically significant (p < 0.05).

**Table 3:** Analysis of QoL in the sample (WHOQoL score)

	*Q1 mean score (SD)*	*Q2 mean score (SD)*	D1 *mean score (SD)*	D2 *mean score (SD)*	D3 *mean score (SD)*	D4 *mean score (SD)*
**Men** **< 45 years**	2.22 (0.89)	2.19 (0.95)	21.75 (5.33)	13.85 (4.48)	4.00 (2.62)	16.77 (5.26)
**Men** **≥ 45 years**	2.39 (0.89)	2.43 (0.94)	20.30 (6.56)	13.69 (4.93)	4.56 (2.95)	17.78 (5.72)
**Women** **< 45 years**	2.27 (1.16)	2.33 (0.97)	22.53 (5.78)	14.13 (3.97)	5.80 (3.00)	15.93 (5.83)
**Women** **≥ 45 years**	1.95 (1.04)	1.62 (0.94)	16.46 (6.28)	11.67 (4.90)	3.42 (2.83)	15.50 (5.67)
**Men Total recorded range**	2.29 (0.87)	2.29 (0.93)	21.05 (5.87)	13.73 (4.59)	4.17 (3.20)	16.95 (5.64)
**Women Total recorded range**	2.12 (1.09)	1.95 (1.02)	19.1 (6.97)	12.8 (4.82)	4.47 (3.20)	15.87 (5.81)

Q1 = quality of life perception; Q2 = perception of one's own health; D1 = domain 1; D2 = domain 2; D3 = domain 3; D4 = domain 4

**Figure 2:**
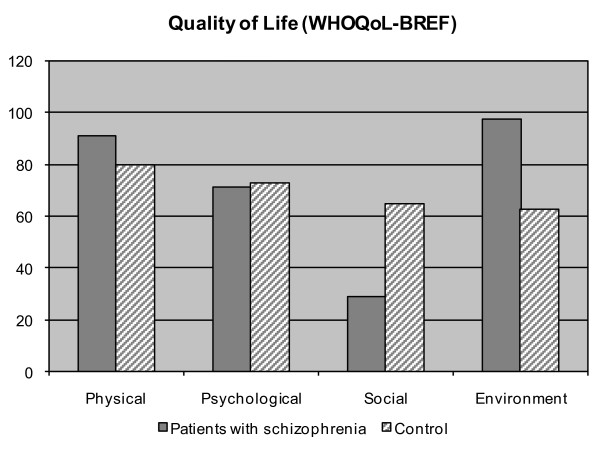
**A survey on the perceived quality of life through WHOQoL-BREF: mean scores obtained in the four domains and comparison with Control of Italian WHOQoL-BREF Validation Group**.

Scores were later turned into 0-100 scale according to specific guidelines, and compared to average of the national Italian WHOQoL validation group [23]. The comparison showed that sample subjects had a lower degree of satisfaction in social relations and psychological domain; however, greater satisfaction in environmental and physical domain emerged. (Figure 2)

### Psychiatric symptoms and global functioning

Average score obtained by symptoms survey through BPRS was 47.3 (SD 18.1; range from 24 to 110). According to the BPRS cut-off score, 12.5% of subjects had a BPRS >72, 27.9% between 49 and 72, and 59.6% ≤48. Table 4 shows average scores obtained by the four subscales characterising BPRS. The total average score obtained in anxiety-depression subscale is 12.88 (SD 5.9), in positive symptoms is 21.02 (SD 5.3), in negative symptoms is 13.41 (SD 7.2) and in mania-hostility 10.39 (SD 5.3).

**Table 4:** Analysis of symptoms (BPRS score) and functioning (FPS score) in the sample

	BPRS anxiety-depression *mean (S.D.)*	BPRS positive *sy*mptoms *mean (S.D)*	BPRS negative symptoms *mean (S.D.)*	BPRS mania-hostility *mean (S.D.)*	BPRS total *mean (S.D.)*
**Men** **< 45 years**	12.6 (6.41)	11.3 (6.42)	12.55 (5.53)	11.31 (5.81)	48.72 (19.86)
**Men** **≥ 45 years**	11.96 (4.79)	9.83 (4.53)	13.96 (7.4)	8.87 (3.15)	44.61 (14.22)
**Women** **< 45 years**	14.87 (6.6)	9.87 (4.8)	11.7 (6.15)	10.86 (4.75)	47.92 (14.92)
**Women** **≥ 45 years**	12.78 (5.92)	8.56 (3.64)	14.52 (8.92)	9.82 (5.29)	46.04 (17.47)
**Total recorded range**	6-30	5-27	7-41	6-29	24-110

	**FPS Scale A** ***mean (S.D.)***	**FPS Scale B** ***mean (S.D.)***	**FPS Scale C** ***mean (S.D.)***	**FPS Scale D** ***mean (S.D.)***	**FPS total** ***mean (S.D.)***

**Men** **< 45 years**	1.75 (1.49)	1.74 (1.11)	1.24 (1.23)	0.62 (1.24)	57.75 (20.82)
**Men** **≥ 45 years**	2.28 (1.66)	2.0 (1.39)	2.04 (1.47)	0.52 (1.01)	52.35 (24.08)
**Women** **< 45 years**	1.07 (1.64)	2.03 (1.39)	1.5 (1.05)	1.1 (1.5)	53.87 (23.01)
**Women** **≥ 45 years**	1.87 (1.52)	2.02 (1.38)	1.56 (1.37)	0.69 (1.27)	56.65 (22.14)
**Total recorded range**	0-4	0-4	0-4	0-4	11-100

Average scores obtained by Personal and Social Functioning Scale are presented in Table 4; total average score is 55.35 (SD 22.4), ranging from 11 to 100. 31.7% of subjects had severe or very severe social difficulties (FPS ≤ 40), 24% had marked difficulties (FPS 41-60), 34.6% had plain or light difficulties (FPS 61-80) and 9.6% had very light or no difficulties at all (FPS >80). In the 4 domains examined by FPS, most difficulties were found in work and socio-relational functioning (difficulty degree mostly between light and plain), followed by self-care and health domain, and finally in disturbed and aggressive behaviours, where only light or no difficulties at all are recorded.

### Variables associated with QoL in patients with schizophrenia

Correlations between WHOQoL, BPRS and FPS are shown in table 5.

**Table 5:** Correlation between global functioning, symptoms and Quality of life (n = 104)

	Q 1	Q 2	WHOQoL domain 1	WHOQoL domain 2	WHOQoL domain 3	WHOQoL domain 4
**Age in 2008**	-0.04	-0.09	-0.35**	-0.21*	-0.12*	-0.08
**Disease length**	0.19*	0.07	-0.18*	-0.06*	0.04	-0.05
**FPS total**	0.28*	0.30*	0.57**	0.34*	0.37*	0.43**
**BPRS anxiety-depression**	-0.32*	-0.29*	-0.08	-0.28*	-0.15*	-0.05
**BPRS positive symptoms**	-0.17*	-0.16*	-0.08	-0.10*	-0.15*	-0.10*
**BPRS negative symptoms**	-0.06	-0.13*	-0.29*	-0.08	-0.10*	-0.16*
**BPRS mania-hostility**	-0.22*	-0.25*	-0.06	-0.13*	-0.14*	-0.03
**BPRStot**	-0.25*	-0.28*	-0.20*	-0.20*	-0.19*	-0.12*

*p < 0.05 **p < 0.0005

Q1 = Overall perception of Quality of Life; Q2 = Overall perception of Health

WHOQoL results related to FPS total score and subscales, especially health and environment satisfaction scales. Personal and social functioning is related negatively to the "health satisfaction" -WHOQoL domain 1 (r = 0.57; p < 0.0005), to the "social relationships satisfaction" -WHOQoL domain 3 (r = 0.37; p < 0.05) and to the"environment satisfaction" -WHOQoL domain 4 (r = 0.43; p < 0.0005).

Patients' age was also negatively correlated to QoL, particularly to "health satisfaction" (r = - 0.35; p < 0.0005) and to "psychological health satisfaction" (r = - 0.12; p < 0.05); similarly disease length was negatively correlated to "health satisfaction" (r = - 0.18; p < 0.05).

Global functioning in our subjects (FPS tot) resulted negatively related to symptoms (BPRS tot), in particular to negative symptoms with a marked negative correlation (r = - 0.65; p < 0.0005).

Significant correlations between negative symptoms (BPRS negative symptoms) and "physical area"-WHOQoL domain 1 (r = 0.29; p < 0.05) and "environment domain"-WHOQoL domain 4 (r = 0.16; p < 0.05) were found; general psychopathology (BPRS anxiety-depression) was related to "psychological domain"-WHOQoL domain 2 (r = - 0.28; p < 0.05), to the"social domain"-WHOQoL domain 3 (r = - 0.15; p < 0.05) and to the individual's overall perception of QoL -Q1 (p = -0.32; p < 0.05); also positive symptoms (BPRS positive symptoms) had correlation to "social domain"-WHOQoL domain 3 (r = - 0.15; p < 0.05) and individual's overall perception of QoL (p = - 0.17; p < 0.05).

## Discussion

In the present study we examined all the subjects with a diagnosis of schizophrenia attending the same Community Mental Health Centre in Northern Italy. Taking into account schizophrenia outcome variability [24-26], psychiatric symptoms and overall functioning and quality of life were assessed in order to verify the relationship between these variables and quality of life.

First, findings obtained through self-administered WHOQoL-BREF suggest that quality of life is not extremely negative, though schizophrenia is often an impairing chronic illness. In some respects, the population studied obtained higher scores on environment and physical dimensions of the WHO-QoL-Bref than average of the national Italian WHOQoL validation group.

With aging, perceived physical health quality gets worse, probably in relation to the increase of such diseases as hypertension, diabetes and rheumatic diseases, that were recorded in our analysis. Curiously enough, as the disease length increases, perceived QoL does not get worse, on the contrary, overall QoL gets better. This could be caused by an increased knowledge of the disease and its treatment possibilities.

In WHOQoL single items scoring, major insatisfactions regard sexual life (absolutely nonsatisfying for 59.7% of males and 54.7% of females), working life (54.8% of males and 50% of females are totally unsatisfied of their working life);

The relation with the health department, including mental health service, is considered negatively only by 12.9% of males and 0.5% of females, a flattering result for the staff members; over 70% of the subjects declare to be satisfied with the environments where they live and of the facilities that are provided.

In our study the psychiatric symptoms and overall functioning resulted diversified, as one third of subjects showed severe or extremely severe functioning difficulties. Consistently with literature [25,27], this suggests that schizophrenia is an extremely heterogeneous illness with many possible variables influencing psychopathology and disability.

Regarding the association of psychiatric symptoms and global functioning to QoL, we found a statistically significant correlation between BPRS and quality of life. Literature shows a great variability among studies on symptoms and QoL in schizophrenia, mostly due to wide variations in measurement strategies and definitions of QoL. Fitzgerald et al. [10], in a study comparing subjective to observer-rated QoL in schizophrenia, showed how subjective reported life satisfaction, measured with "SCAP instrument" (which is a 100-item self-report instrument that contains a number of items of self-report life satisfaction) was not related to positive or negative symptoms, but was correlated with depressive symptoms. Ross et al. [11], in a study on QoL, symptoms and level of functioning in schizophrenia, suggest that QoL in schizophrenia is more highly related to negative rather than positive symptoms. Weighted effect size analyses revealed small relationships between psychiatric symptoms and QoL, with general psychopathology showing the strongest negative associations across all QoL indicators [4].

In our study, we found a statistically significant correlation between symptoms and quality of life. More symptomatic subjects have a worse perception of QoL. Especially anxiety-depression symptoms determine a worse evaluation of general QoL, satisfaction own health and psychological health, whilst negative symptoms are associated with a bad perception of health satisfaction. These results agree with what reported by other authors [10,11,28].

Major statistically significant correlations of QoL regarded overall functioning; most correlations were found between all subscales of FPS and WHOQoL domain 1 (physical area), and domain 4 (environment), while lower, even if statistically significant, correlations were shown between FPS tot and WHOQoL domains 2 and 3 (psychological and social). As the level of personal and social disability increases, the dissatisfaction about physical health, environment, social and eventually psychological aspects increases, in an overall unsatisfaction about QoL.

These results point out how personal and social functioning plays a key role, in schizophrenia, in determining subjects' quality of life, while symptoms, though related to QoL, do not seem to be the major variable in molding QoL.

Psychiatric symptoms also were found to be negatively associated with overall functioning. The results of a cross-sectional study using data from a large study [29] suggested similarly that symptoms may be strongly related to functioning [30]. According to our data, a major highly significant correlation is related to negative symptoms and functioning measured through FPS (r = 0.65; p < 0.0005). These findings confirm the well-documented strong relationship between negative symptoms and social disability [31-33].

Certain limitations of the study should be mentioned. First, the study was cross-sectional; therefore, the exploration of causal relationship was rather tentative. Second, a relatively limited number of variables was examined; in addition to socio-demographic and clinical data, a complex interaction of other factors, such as self-esteem, premorbid adjustment, therapy and social support network could also play a role in determining subjective quality of life.

Finally, a potential limitation of the study was the use of the WHOQOL-BREF, a generic questionnaire that may not have detected subtle changes of subjective QoL in the specific population of schizophrenia patients.

## Conclusions

The World Health Organisation [34] reports that there has been a worldwide pattern shift from hospital care to community-based care of people with mental illnesses. Italy has worked on decentralising its mental health services since 1978. The present research on social functioning, psychiatric symptoms and QoL in people with schizophrenia suggests that symptoms, but, above all, personal and social functioning are important elements to determine QoL. These studies point to the importance of looking beyond symptom-reduction strategies for improving QoL in schizophrenia; furthermore, they underline how rehabilitation facilities and increased participation of families and communities in the treatment significantly improve quality of life of people with mental illness.

## Competing interests

The authors declare that they have no competing interests.

## Authors' contributions

All authors participated in the design of the study. AG, MCT, MGN and PM wrote the manuscript. MCT and LG reviewed the manuscript. MCT performed the statistical analysis. All authors read and approved the final manuscript.
